# Shenmai Injection Supresses Glycolysis and Enhances Cisplatin Cytotoxicity in Cisplatin-Resistant A549/DDP Cells via the AKT-mTOR-c-Myc Signaling Pathway

**DOI:** 10.1155/2020/9243681

**Published:** 2020-06-20

**Authors:** Ye Sun, Yushi Chen, Ming Xu, Chunying Liu, Hai Shang, Chun Wang

**Affiliations:** ^1^Department of Cell Biology, College of Integrated Chinese and Western Medical, Liaoning University of Traditional Chinese Medicine, 79 Chongshan Eastern Road, Huang gu District, Shenyang 110847, China; ^2^Key Laboratory of Environmental Pollution and Microecology of Liaoning Province, Shenyang Medical College, Shenyang 110034, China; ^3^Liaoning Cancer Hospital & Institute, Shenyang 110042, China

## Abstract

Tumor cells, especially drug-resistant cells, predominately support growth by glycolysis even under the condition of adequate oxygen, which is known as the Warburg effect. Glucose metabolism reprogramming is one of the main factors causing tumor resistance. Previous studies on Shenmai injection (SMI), a Chinese herbal medicine, have shown enhanced efficacy in the treatment of tumors in combination with chemotherapy drugs, but the mechanism is not clear. In this study, we investigated the effect of SMI combined with cisplatin on cisplatin-resistant lung adenocarcinoma A549/DDP cells. Our results showed that cisplatin-resistant A549/DDP cells exhibited increased glucose consumption, lactate production, and expression levels of key glycolytic enzymes, including hexokinase 2 (HK2), pyruvate kinase M1/2 (PKM1/2), pyruvate kinase M2 (PKM2), glucose transporter 1 (GLUT1), and lactate dehydrogenase A (LDHA), compared with cisplatin-sensitive A549 cells. SMI combined with cisplatin in A549/DDP cells, led to significantly lower expression levels of key glycolytic enzymes, such as HK2, PKM1/2, GLUT1, and pyruvate dehydrogenase (PDH). In addition, we found that the combination of SMI and cisplatin could inhibit cell proliferation and promote apoptosis by reducing the expression levels of p-Akt, p-mTOR, and c-Myc, and then, it reduced the glycolysis level. These results suggest that SMI enhances the antitumor effect of cisplatin via glucose metabolism reprogramming. Therefore, the combination of SMI and cisplatin may be a potential therapeutic strategy to treat cisplatin-resistant nonsmall cell lung cancer.

## 1. Introduction

The antitumor activities of cisplatin, such as induction of DNA damage and mitochondrial apoptosis, have been widely used in chemotherapy for many kinds of tumors, especially for advanced lung cancer [1]. Long-term cisplatin treatment partially leads to a variety of glucose metabolic pathways, including the glycolysis level and the expression of key enzymes, resulting in poor treatment with cisplatin, but the precise cisplatin resistance mechanism has not been completely understood [2, 3].

Shenmai injection (SMI) is derived from Shengmai San, the well-known Chinese medicine prescription, which consists of Radix Ginseng Rubra and Radix Ophiopogonis [4]. SMI is used to improve myocardial function and enhance immunity; recently, it has been found to increase the therapeutic effect combined with chemotherapy drugs in antitumor treatment [5, 6]. Recently, Liu reported that SMI enhances the cytotoxicity of chemotherapy drugs against colorectal cancer by improving the distribution of drugs in cells [7]. SMI has an obvious inhibitory effect on various tumors in mice, which effectively prolongs the survival time of tumor-bearing mice [8]. However, the exact antitumor mechanism of SMI is still unknown.

In this study, we first evaluated the difference in glycolysis metabolism between cisplatin sensitive cells (human lung adenocarcinoma cell line A549) and cisplatin-resistant cells (A549/DDP cells), and subsequently, we explored the antitumor mechanism of SMI in reversing cisplatin resistance in A549/DDP cells.

## 2. Materials and Methods

### 2.1. Cell Lines and Cell Culture

Human lung adenocarcinoma cell line (A549) was purchased from the Beijing Dingguo Changsheng Biotechnology Company (Beijing, China). Human lung adenocarcinoma cisplatin-resistant cell line (A549/DDP) was purchased from the Cancer Hospital of Chinese Academy of Medical Sciences (Beijing, China). The cells were cultured in Dulbecco's Modified Eagle Medium/High Glucose (DMEM/High Glucose) (Hyclone, Logan, UT, USA) containing 10% fetal bovine serum (Scitecher, Oxford, MS, USA), 100 U/mL penicillin, and 100 mg/mL streptomycin (Genview, Australia), and they were cultivated at 37°C in a 5% CO_2_ incubator. The A549/DDP cell medium contained 16.7 *μ*M cisplatin to maintain drug resistance for 2 weeks, and no cisplatin for 2 weeks before the experiment, the stability of cisplatin resistance in A549/DDP cells can be maintained for one month.

### 2.2. Antibodies and Reagents

SMI (the concentration of crude drug was 200 mg/ml) was obtained from Shineway Pharmaceutical Co., Ltd. (Hebei, China). Cisplatin was purchased from Sigma Aldrich Company (St. Louis, MO, USA). The antibodies used in the experiment are shown in Table 1.

### 2.3. Inhibition Assay of SMI and Cisplatin

Inhibition rate assay of SMI alone and cisplatin alone is in A549/DPP cells. Briefly, 5 × 103 A549/DDP cells were seeded into a 96-well plate and cultured for 12 h. Then the cell culture medium was discarded and treated with various concentrations of SMI (4, 8, 16, 32, 64, and 128 mg/ml) or cisplatin (12, 24, 48, 96, and 192 *μ*M) for 24 h. Further, 10 *μ*L of cell counting kit-8 solution was added (Apexbio, Houston, TX, USA) to each plate, and the plate was incubated for 4 h. The absorbance of each individual experiment at 460 nm was detected at least three times. IC_5_, IC_10_, IC_20_, and IC_50_ values were calculated via using linear regression on the inhibition rate curve of SMI or cisplatin. Inhibition rate = (OD_contol_ − OD_experiment_)/(OD_contol_ − OD_blank_) × 100%.

Inhibition rate of SMI combined with cisplatin in cisplatin-resistant A549/DPP cells. Briefly, 5 × 103 A549/DDP cells were seeded into a 96-well plate and cultured for 12 hours. The cell culture medium was discarded, and combined cisplatin and different concentrations of SMI at concentrations of 10 mg/mL, 15 mg/mL, and 20 mg/mL (equivalent to IC_5_, IC_10_, and IC_20_) were added to the culture for 24 h. Further, 10 *μ*L of cell counting kit-8 solution was added to each plate, and the plate was incubated for 4 h. The measurement and calculation method was the same as above, no SMI as the control.

### 2.4. Glucose Consumption and Lactate Production Assay

Briefly, 2 × 105 A549/DDP cells were seeded into 6-well plates and cultured for 24 h. The culture medium and cells were collected separately, and glucose concentration and lactate production were measured using the Glucose assay kit (Applygen, Beijing, China) and the Lactic Acid assay (Jiancheng Bioengineering Institute, Nanjing, China) in accordance with the manufacturers' protocols, and the glucose consumption level or the lactate production level was divided by the number of cells.

### 2.5. 5-Ethynyl-2′-Deoxyuridine (EdU) Assay

Briefly, 2.5 × 104 A549/DDP cells were seeded into a 24-well plate. SMI at concentrations of IC_5_, IC_10_, and IC_20_ was combined with the cisplatin concentration of IC_20_ to treat A549/DDP cells for 24 h, and then, 500 *μ*L 1×EdU solution (final concentration 10 *μ*M) was added and incubated for 2 hours. Phosphate buffered saline (PBS) was used to wash the cells and 4% paraformaldehyde was used to fix the cells for 30 min. EdU staining and DNA staining were performed by the EdU Kit (Donghuan Biotech, Shanghai, China), and images were acquired by fluorescence microscopy (Zeiss, Germany).

### 2.6. Inhibitor Assay

Briefly, 2 × 105 A549/DDP cells were seeded on 6-well plates to adhere well. LY294002 (phosphorylated Akt inhibitor; Abmole Bioscience, Shanghai, China; 20 *μ*M), rapamycin (phosphorylated mTOR inhibitor; Abmole Bioscience, Shanghai, China; 5 nM), and 10058-F4 (c-Myc inhibitor; Abmole Bioscience, Shanghai, China; 20 *μ*M) were added to the cells for 12 h, and the same volume of dimethyl sulfoxide (DMSO) was used as the control. Then, the culture medium was removed, and mixed drugs containing 20 mg/ml SMI and 23.3 *μ*M cisplatin were added, and 10% fetal bovine serum DMEM was used as a control and cultured for 24 h. Finally, the cells were collected and total proteins were extracted. The expression levels of related proteins were probed by western blot.

### 2.7. Real-Time PCR (qRT-PCR)

RNA was isolated with RNAiso plus (Takara, Dalian, China), and cDNA was reverse transcribed by Reverse Transcription Kit (Takara, Dalian, China). The expression of the target gene was quantitated by using SYBR Green Master Mix (Vazyme, Nanjing, China) according to the manufacturer's protocol. The primers used in the study are shown in Table 2.

### 2.8. Western Blot Analysis

Total cellular proteins were isolated with RIPA Lysis buffer (Beyotime, China) with a final phenylmethanesulfonyl fluoride (PMSF) concentration of 1 mM (Beyotime, China). Protein concentration was measured by the BCA protein assay (Leagene, Beijing, China) according to the manufacturers' protocols to ensure equal protein loading in each sample. The protein extracts (50 *μ*g) were separated by 8–10% SDS-polyacrylamide gel electrophoresis and then transferred to a polyvinylidene fluoride membrane (Millipore) at 300 mA for 2 h. The membrane was blocked with skimmed milk overnight, and then, it was maintained with the primary antibody for 1 h at room temperature and with the secondary antibody for 1 h at room temperature. The membranes were maintained with enhanced chemiluminescence western blot detection kit (Vazyme, Nanjing, China) and detected using bio-imaging systems (DNR, Israel). The expression of *β*-actin was used as the internal control.

### 2.9. Statistical Analyses

All of the experiments were repeated three times, and the data are expressed as mean ± SD. A two-tailed Student's *t*-test was used to assess the differences between the two groups. An analysis of variance (ANOVA) test was used to assess the differences between multiple sets of data. *P* < 0.05 was considered to be significant. Data were analyzed using SPSS 19.0.

## 3. Results

### 3.1. A549/DDP Cells Exhibit Increased Aerobic Glycolysis

**Figure 1:**
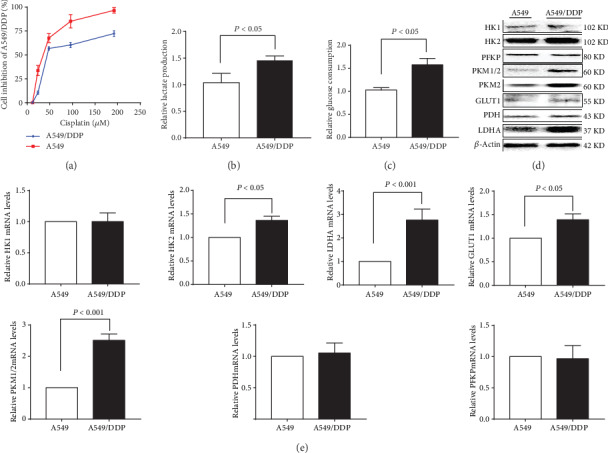
Glucose metabolism was upregulated in cisplatin-resistant A549/DDP cells. (a) Detection of cisplatin inhibition in cisplatin-sensitive or cisplatin-resistant A549 cells and A549/DDP cells. (b) Glucose consumption and (c) lactate production were measured in cisplatin-sensitive or cisplatin-resistant A549 cells and A549/DDP cells. Expression of glucose metabolism enzymes at the protein level or the mRNA level in cisplatin-sensitive or cisplatin-resistant A549 and A549/DDP cells by (d) western blot and (e) RT-PCR analysis. The results are presented as mean ± S.D. (*n* = 3); the experimental results are presented in triplicate. HK1, hexokinase 1; HK2, hexokinase 2; PFKP, platelet-type phosphofructokinase; PKM1/2, Pyruvate Kinase M1/2; PKM2, pyruvate kinase M2; GLUT1, glucose transporter 1; PDH, pyruvate dehydrogenase; LDHA, lactate dehydrogenase A.

First, we measured the inhibition curves of A549 and A549/DDP cells at different concentrations of cisplatin, and the results showed that IC_50_ of A549 and A549/DDP to cisplatin were 37.8 *μ*M and 63.3 *μ*M, respectively. It has been reported that the aerobic glycolytic activity of cancer cells is abnormally elevated compared with that of normal cells, especially of drug-resistant cancer cells, which has become one of the main mechanisms of drug resistance [9]. In this study, we assessed the level of glucose metabolism, as shown in Figure 1(b); glucose consumption in A549/DDP cells was increased compared with that in A549 cells (*P* < 0.05). A549/DDP cells showed a similar increased trend in lactate production compared to A549 cells (*P* < 0.05), in Figure 1(c). Then, we analyzed the expression levels of key glycolytic enzymes at the protein and mRNA levels. The mRNA expression levels and protein expression levels of hexokinase 2 (HK2), pyruvate kinase M1/2 (PKM1/2), pyruvate kinase M2 (PKM2), glucose transporter 1 (GLUT1), and lactate dehydrogenase A (LDHA) were increased on comparison of A549/DDP cells with A549 cells (Figures 1(d) and 1(e)).

### 3.2. SMI Enhances Cisplatin Cytotoxicity in A549/DDP Cells

SMI and cisplatin have direct inhibition on A549/DDP cell growth, and the IC_50_ of SMI was 40.00 mg/mL, and the IC_5_, IC_10_, and IC_20_ were 10.0, 15.0, and 20.0 mg/mL, respectively; meanwhile, the IC_50_ of cisplatin was 63.6 *μ*M, and IC_5_, IC_10_, and IC_20_ were 6.6, 12.3, and 23.3 *μ*M, respectively.

**Figure 2:**
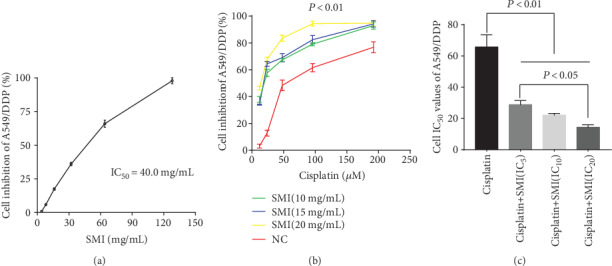
SMI enhances the inhibition of cisplatin in the proliferation of cisplatin-resistant A549/DDP cells. (a) Determination of cisplatin-resistant A549/DDP cell inhibition rate curve with SMI. (b) Inhibition rate curve of SMI at concentrations of IC_5_, IC_10,_ and IC_20_ combined with cisplatin in cisplatin-resistant A549/DDP cells. (c) Comparison of cisplatin's IC_50_ combined with SMI at concentrations of IC_5_, IC_10,_ and IC_20_ in cisplatin-resistant A549/DDP cells. The results represent the mean ± S.D. (*n* = 3); the experimental results are presented in triplicate. SMI: Shenmai injection; IC: inhibitory concentration.

Cotreatment with SMI and cisplatin interfered with A549/DDP cells; with the increase in concentration of SMI, the inhibition rate of A549/DDP cells increased significantly (Figure 2(b)). By cotreatment with SMI at concentrations of 10.0, 15.0, and 20.0 mg/mL, the IC_50_ values of cisplatin were decreased from 63.6 *μ*M to 21.0 *μ*M, 20.3 *μ*M, and 13.7 *μ*M, respectively (Figure 2(c)).

### 3.3. Combination of SMI and Cisplatin Suppresses A549/DDP Cell Glycolysis and Proliferation

**Figure 3:**
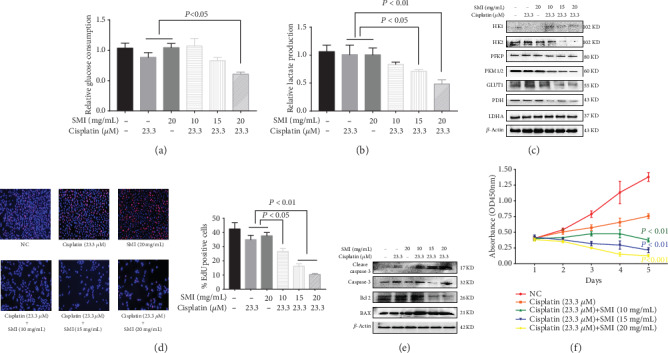
Combination of SMI and cisplatin suppresses A549/DDP cell glycolysis and proliferation and promotes apoptosis. (a) Glucose consumption and (b) lactate production were measured with SMI at concentrations of IC_5_, IC_10_, and IC_20_ combined with cisplatin (IC_20_) in cisplatin-resistant A549/DDP cells. (c) Expression levels of glycometabolic key enzymes were measured with SMI at concentrations of IC_5_, IC_10_, and IC_20_ combined with cisplatin (IC_20_) in cisplatin-resistant A549/DDP cells by western blot analysis. (d) Edu assay analyzed the proliferation of cisplatin-resistant A549/DDP cells with SMI at concentrations of IC_5_, IC_10_, and IC_20_ combined with cisplatin (IC_20_) for 24 h. (e) Western blot measured the expression of apoptosis-related proteins of cisplatin-resistant A549/DDP cells with SMI at concentrations of IC_5_, IC_10_, and IC_20_ combined with cisplatin (IC_20_) for 24 h. (f) The effects of SMI at concentrations of IC_5_, IC_10_, and IC_20_ combined with cisplatin (IC_20_) on the growth of cisplatin-resistant A549/DDP cells were measured at 1, 2, 3, 4, and 5 days, respectively. The results are expressed as the mean ± S.D. (*n* = 3); the experimental results are presented in triplicate. NC: negative control; SMI: Shenmai injection.

Cotreatment with SMI and cisplatin weakened A549/DDP cell glucose consumption and lactate production (Figures 3(a) and 3(b)). Notably, in the group of 20 mg/mL SMI combined with cisplatin (23.3 *μ*M), glucose consumption was significantly decreased compared with that in the cisplatin alone group and the SMI alone group (*P* < 0.05). Similarly, the lactate production was significantly decreased in the group of SMI 15 mg/mL or 20 mg/mL combined with cisplatin 23.3 *μ*M compared with the cisplatin alone group and the SMI alone group (*P* < 0.05). With the increase in SMI concentration, the expression levels of key glycolytic enzymes in A549/DDP cells were significantly inhibited, including HK2, PKM1/2, GLUT1, and PDH rather than HK1, PFKP, and LDHA, compared with the cisplatin alone group and the SMI alone group (Figure 3(c)).

The proliferation of A549/DDP cells was significantly inhibited with the increase in concentration of SMI with the same cisplatin concentration after 24 h (Figure 3(d)). In addition, the expression levels of apoptosis-related proteins, such as Caspase 3, cleaved Caspase 3, and Bcl 2, were significantly increased, and the expression level of BAX was significantly decreased in the group of SMI combined with cisplatin 23.3 *μ*M compared with the cisplatin alone group and the SMI alone group (Figure 3(e)). The proliferation of A549/DDP cells was also significantly inhibited after treatment of SMI combined with cisplatin for 5 days (Figure 3(f)).

### 3.4. SMI Enhances Cisplatin-Suppressed Expression Levels of P-AKT, P-mTOR, and c-Myc

**Figure 4:**
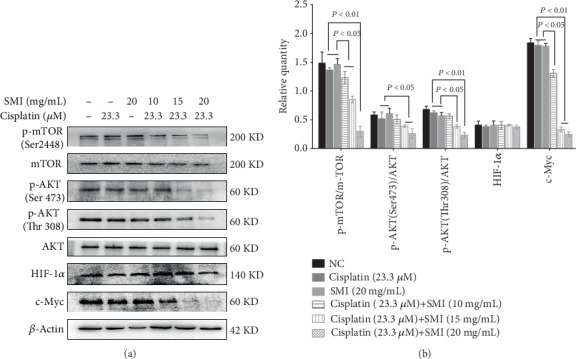
Combination of SMI and cisplatin inhibition effect on expression levels of p-AKT, p-mTOR, and c-Myc in cisplatin-resistant A549/DDP cells and HIF-1*α* in cisplatin-resistant A549/DDP cells. (a) Western blot analysis of protein expression levels of AKT, p-AKT, mTOR, p-mTOR, c-Myc, and HIF-1*α*. (b) Quantification of relative protein expression levels of p-AKT, p-mTOR, c-Myc, and HIF-1*α*. The results represent the mean ± S.D. (*n* = 3); the experimental results are presented in triplicate. AKT: AKT serine/threonine kinase; mTOR: mechanistic target of rapamycin; p: phosphorylated; HIF-1*α*: hypoxia-inducible factor-1*α*; c-Myc: cellular-myelocytomatosis viral oncogene.

Numerous studies have suggested that AKT/mTOR is involved in the Warburg effect by activating HIF-1*α* or c-Myc in tumor cells. Our results showed that cotreatment with SMI and cisplatin significantly inhibited the expression levels of p-AKT (Ser473), p-AKT (Thr308), p-mTOR (Ser2448), and c-Myc in A549/DDP cells; further, the higher the concentration of SMI, the more obvious the inhibition. However, HIF-1*α* expression levels did not change significantly (Figures 4(a) and 4(b)).

### 3.5. SMI Enhances the Inhibitory Effect of Cisplatin on Glucose Metabolism via the AKT-mTOR-c-Myc Pathway

**Figure 5:**
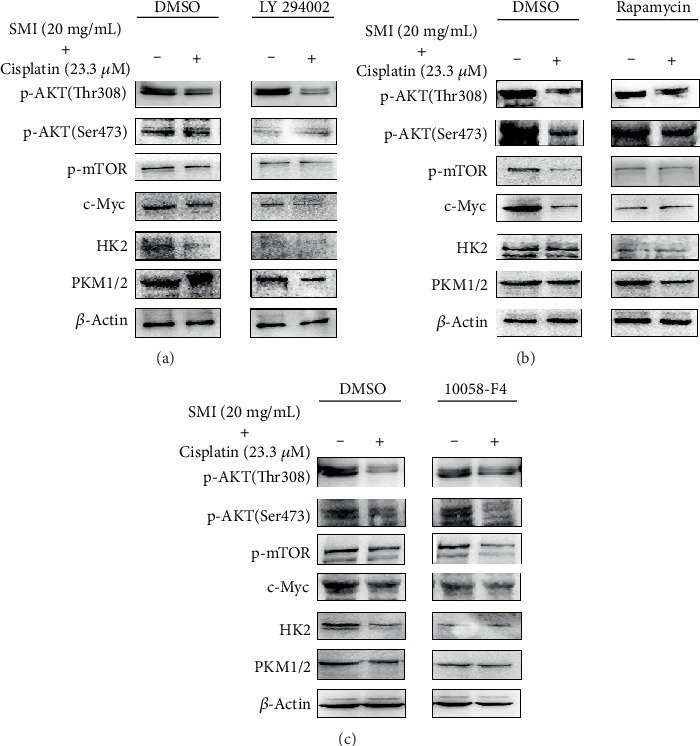
SMI enhances cisplatin cytotoxicity in cisplatin-resistant A549/DDP cells via the AKT-mTOR-c-Myc signaling pathway. (a) LY294002 (PI3K inhibitor), (b) rapamycin (mTOR inhibitor), and (c) 10058-F4 (c-Myc inhibitor) were treated or not treated in cisplatin-resistant A549/DDP cells via western blot analysis of protein expression levels of genes involved in cell glucose metabolism.

After pretreatment with LY294002 (inhibitor of PI3K), A549/DDP cells were incubated with the mixed drugs (SMI 20 mg/mL + cisplatin 23.3 *μ*M). In these cells, the expression levels of p-AKT (Ser473), p-mTOR, c-Myc, HK2, and PKM1/2 were significantly reduced compared with those in the single mixed drug treatment group and the LY294002 alone treatment group, but the expression of p-AKT (Thr308) was not changed (Figure 5(a)). Similarly, after treatment with rapamycin (mTOR inhibitor) and mixed drugs (SMI 20 mg/mL + cisplatin 23.3 *μ*M), the expression levels of p-mTOR, c-Myc, HK2, and PKM1/2 in A549/DDP cells were significantly lower than those in the mixed drug treatment group and the rapamycin treatment group alone, respectively, and the expression levels of p-AKT (Ser473) and p-AKT (Thr308) remained unchanged (Figure 5(b)). Finally, A549/DDP cells were incubated with 10058-F4 (c-Myc inhibitor) and mixed drugs successively; the expression levels of c-Myc, HK2, and PKM1/2 were significantly reduced compared to those in the mixed drug treatment group alone and the 10058-F4 treatment group alone, but the expression levels of p-AKT (Ser473), p-AKT (Thr308), and p-mTOR were not changed (Figure 5(c)). It was concluded that SMI combined with cisplatin suppressed the expression level of cell glucose metabolism genes through the AKT-mTOR-c-Myc signaling pathway.

## 4. Discussion

Numerous studies have shown that most cancer cells exhibit increased glycolysis even in the presence of oxygen, including increased glucose consumption and lactate production [10, 11]. Abnormal metabolic shifts in cancer cells are frequently related to tumor growth and increased drug resistance, in which some rate-limiting enzymes directly regulate the glycolysis level to promote drug resistance of cancer cells [12]. HK2 is the first rate-limiting enzyme in the glycolysis process, which promotes cisplatin resistance by enhancing drug-induced and ERK-mediated autophagy in ovarian cancer [13]. HK1 is also a member of the hexokinase family, and it has been found to be related to cell activity and proliferation [14]. The second rate-limiting enzyme in the glycolysis process is phosphofructokinase (PFK), which is responsible for the phosphorylation of fructose-6-phosphate to fructose-1,6-diphosphate. A study showed that rectal cancer cells, highly resistant to 5-fluorouracil and oxaliplatin, have enhanced glucose uptake and significantly higher levels of lactic acid due to increased activity of HK and PFK [15]. In addition, pyruvate kinase (PK) is the last rate-limiting enzyme in the glycolysis process, which is involved in the conversion of phosphoenol pyruvate into pyruvate. Cheng et al. found that the expression of PKM2 was increased in two drug-resistant colon cancer cell lines, vincristine resistant HCT-8 cell line, and oxaliplatin resistant HCT116 cell line [16]. In addition, it was found that some enzymes are also involved in the development of drug resistance. For example, GLUT1 is responsible for regulating glucose transport inside and outside the cells, and it has been found to be upregulated in insulin-resistant liver cancer cells [17]. It was found that lactate dehydrogenase (LDH) activity was enhanced in tumor cells to produce a large amount of lactic acid, which placed the tumor cells in an acidic microenvironment to promote their growth. Studies have shown that the effects of chemotherapeutic drugs can be enhanced by inhibiting the activity of LDH [18, 19]. In this study, we also found increased glucose consumption and lactic acid accumulation, as well as increased expression levels of HK1, HK2, PKM1/2, PKM2, GLUT1, and LDHA in cisplatin-resistant A549/DDP cells compared to A549 cells, which suggested that glycolysis induced drug resistance in nonsmall cell lung carcinoma cells.

In the traditional Chinese medicine (TCM) theory, *qi* is considered to be the basic substance of human activities and life energy, including physical and spiritual levels. We have shown that some TCMs with the functionality of invigorating *qi*, such as Bu-Zhong-Yi-Qi decoction and Shenqi Fuzheng injection, might increase chemotherapy sensitivity in cisplatin resistance of NSCLCs by activating apoptosis and the autophagy pathway and inducing cell cycle arrest [20]. SMI is the precise TCM, which has the function of invigorating *qi*. Previous studies have found that SMI combined with chemotherapeutic drugs improves the clinical efficacy and relieves the adverse reactions, including incidence of leukopenia and gastrointestinal reactions in gastric and breast cancer patients [21]. At present, most of the studies on SMI are based on a network meta-analysis, and its antitumor mechanism is still unclear. Our results showed that SMI combined with cisplatin reduced the glucose consumption and lactate production in A549/DDP cells by inhibiting the expression levels of key glycolytic enzymes, including HK2, PKM1/2, GLUT1, and PDH, thereby enhancing the toxic effect of cisplatin on A549/DDP cells. Furthermore, we detected the expression levels of Caspase 3, Bcl 2, and BAX by Edu proliferation and western blot, and the results showed that SMI could enhance the inhibitory effect of cisplatin on A549/DDP cell proliferation and promote cell apoptosis.

Previous studies have found that the AKT/mTOR signaling pathway is one of the most important signaling pathways in cancer, and it plays an important role in cell proliferation and survival. The Akt/mTOR pathway is involved in regulating the expression of HIF-1*α*. Recent research has found that HIF-1*α* is activated via the Akt/mTOR pathway, enhancing the migration and invasion of human glioblastoma U87 cells [22]. Furthermore, the AKT/mTOR signaling pathway can also regulate c-Myc to affect the metabolism and growth of cancer cells [23]. Under hypoxic conditions, HIF-1*α* mainly regulates the glycolysis-related target genes, whereas c-Myc regulates the same genes under normoxic conditions [11, 24]. In this study, we conducted experiments under normoxic conditions, and we found that the combination of SMI and cisplatin downregulated the levels of p-AKT (Ser473), p-AKT (Thr308), p-mTOR (Ser2448), and c-Myc of the AKT/mTOR/c-Myc signaling pathway in A549/DDP cells, but the expression of HIF-1*α* was not changed. Reduced AKT/mTOR/c-Myc signaling pathway suppressed the expression levels of key enzymes, such as HK1, HK2, PKM1/2, PKM2, GLUT1, and PDH, in the glycolysis pathway. In addition, we also interfered with several key points of the Akt/mTOR/c-Myc signaling pathway by adding inhibitors. The results showed that inhibition of Akt phosphorylation was inhibited by adding LY294002, and there was inhibition of the expression of p-Akt, p-mTOR, c-Myc, and key enzymes of glucose metabolism by SMI combined with cisplatin; mTOR phosphorylation was inhibited by adding rapamycin, and SMI combined with cisplatin could still reduce the expression of p-Akt, but it could not reduce the expression of p-mTOR, c-Myc, and key enzymes of glucose metabolism; the same 10058-f4 could inhibit the expression of c-Myc; SMI combined with cisplatin could still reduce the expression of p-Akt and p-mTOR, but it could not reduce the expression of c-Myc and key enzymes of glucose metabolism in the pathway. In conclusion, these results suggest that SMI is through Akt/mTOR/c-Myc signaling pathway sensitized cisplatin to cisplatin-resistant A549/DDP cells.

Recently, a new study confirmed that SMI can inhibit the function and expression of P-gp through the MAPK/NF-*κ*B signaling pathway and enhance the sensitivity of breast cancer cells to chemotherapy [25]. Combined with our study, SMI can also reprogram glucose metabolism, inhibit cell proliferation, and promote cell apoptosis through the Akt/mTOR/c-Myc signaling pathway, enhance the sensitivity of lung cancer cells to chemotherapy drugs, and improve the mechanism of SMI sensitization towards chemotherapy drugs. However, more in-depth and extensive investigations are needed.

## 5. Conclusions

The present study suggests that the addition of SMI could increase the cytotoxicity of cisplatin by inhibition of the glycolysis metabolism through the AKT/mTOR/c-Myc pathway, which might provide a theoretical basis for the treatment of cisplatin-resistant nonsmall cell lung cancer.

## Figures and Tables

**Table 1:** Antibodies used in the experiment.

Antibody name	Product code	Source	Host	Dilution
HK2	#2867	Cell signaling technology	Rabbit	1 : 1000
PFKP	#5412	Cell signaling technology	Rabbit	1 : 1000
PKM2	#4053	Cell signaling technology	Rabbit	1 : 1000
PKM1/2	#3186	Cell signaling technology	Rabbit	1 : 1000
PDHX	#3205	Cell signaling technology	Rabbit	1 : 1000
AKT	#4691	Cell signaling technology	Rabbit	1 : 500
p-Akt (Thr308)	#2965	Cell signaling technology	Rabbit	1 : 1000
p-Akt (Ser473)	33281 M	Bioss	Mouse	1 : 500
mTOR	#2983	Cell signaling technology	Rabbit	1 : 1000
p-mTOR (Ser2448)	#5536	Cell signaling technology	Rabbit	1 : 1000
HIF-1*α*	#3716	Cell signaling technology	Rabbit	1 : 1000
HK1	ab65069	Abcom	Rabbit	1 : 500
GLUT1	ab652	Abcom	Rabbit	1 : 500
LDHA	ab125683	Abcom	Rabbit	1 : 1000
*β*-Actin	ab 179467	Abcom	Rabbit	1 : 1000
Caspase 3/p17/19	19677-10-AP	Proteintech	Rabbit	1 : 1000
BAX	50599-2-lg	Proteintech	Rabbit	1 : 4000
Bcl 2	26593-1-AP	Proteintech	Rabbit	1 : 1000
Goat anti-rabbit IgG	ZB-2301	Zsbio	Goat	1 : 5000
Goat anti-mouse IgG	ZB-2305	Zsbio	Goat	1 : 5000

**Table 2:** Primer sequences used for RT-PCR.

Gene	Primer	Sequence
HK1	Forward	5′-GCTCTCCGATGAAACTCTCATAG-3′
	Reverse	5′-GGACCTTACGAATGTTGGCAA-3′

HK2	Forward	5′-CCCGTGCCCACAATGAGAC-3′
	Reverse	5′-CCCGTGCCCACAATGAGAC-3′

PFKP	Forward	5′-GCATGGGTATCTACGTGGGG-3′
	Reverse	5′-CTCTGCGATGTTTGAGCCTC-3′

PKM1/2	Forward	5′-ATGTCGAAGCCCCATAGTGAA-3′
	Reverse	5′-TGGGTGGTGAATCAATGTCCA-3′

PDHX	Forward	5′-TTGGGAGGTTCCGACCTGT-3′
	Reverse	5′-CAACCACTCGACTGTCACTTG-3′

GLUT1	Forward	5′-TCATCGTCGCTGAACTCTTCAG-3′
	Reverse	5′-TCACACTTGGGAATCACCCCC-3′

LDHA	Forward	5′-ATGGCAACTCTAAAGGATCAGC-3′
	Reverse	5′-CCAACCCCAACAACTGTAATCT-3′

## Data Availability

The data used to support the findings of this study are available from the corresponding author upon request.
